# Mammoth 2.0: will genome engineering resurrect extinct species?

**DOI:** 10.1186/s13059-015-0800-4

**Published:** 2015-11-04

**Authors:** Beth Shapiro

**Affiliations:** Department of Ecology and Evolutionary Biology and UCSC Genomics Institute, University of California Santa Cruz, Santa Cruz, CA 95060 USA

## Abstract

It is impossible to ‘clone’ species for which no living cells exist. Genome editing may therefore provide the only means to bring extinct species — or, more accurately, extinct traits — back to life.

## Introduction

Coincident with the release of the latest in the ‘Jurassic Park’ series of movies, George Church’s lab at Harvard University’s Wyss Institute reported their first successes in editing living elephant cells so that they contain gene sequences from the elephant’s recently extinct relative, the woolly mammoth [1]. Using a CRISPR (clustered regularly interspaced short palindromic repeats)-Cas9 approach, Church’s team replaced 14 loci in the elephant genome with the mammoth version of those sequences. Although they have not yet created a mammoth, their success blurred the already fuzzy line that separates science from science fiction, bolstering hopes (and fears) that de-extinction, the resurrection of extinct species, may soon be reality.

According to George Church, his team’s goal is to create elephants that have mammoth-derived adaptations to cold climates. Their initial targets for genetic modification include genes that affect blood hemoglobin, ear size, subcutaneous fat, and hair. At present, they are focusing on transforming edited cells into tissues or stem cells to test for altered phenotypes. If the team succeeds in creating genetically engineered elephants, these animals could be introduced into the environments in which mammoths once lived, both expanding the range of habitats in which elephants can live and re-establishing ecological interactions that were lost when mammoths disappeared. This goal — to re-establish interactions between species that were lost as a consequence of extinction and hence to revitalize existing ecosystems — is the stated motivation for most existing de-extinction efforts, including those for passenger pigeons [2], aurochs [3], and American chestnut trees [4].

## Existing technologies

The feasibly of de-extinction varies among organisms, and not all organisms face the same technical challenges in their resurrection [5]. For recently extinct species, it may be possible to use ‘standard’ cloning technology (such as the nuclear transfer followed by cellular reprogramming technique that most famously resulted in the birth of ‘Dolly the Sheep’ in 1996 [6]) and a closely related species as a surrogate maternal host. Cloning via nuclear transfer has been accomplished for a wide range of mammalian species, including several examples in which a species other than that of the developing embryo is used as a surrogate mother [7]. This inter-species nuclear transfer approach is being used to resurrect the bucardo, a subspecies of mountain goat that was endemic to the Pyrenees and went extinct in 2000 [8]. If extinction occurred before living tissues could be collected and preserved, however, cloning is not possible because DNA decay begins immediately after death. The first step to resurrecting long-extinct species is therefore to sequence and assemble a genome from the preserved remains of that extinct species. The past decade has seen enormous advances in technologies for ancient DNA isolation and genome assembly [9], and high-quality genomes are now available for several extinct species, including mammoths and passenger pigeons, while this work is in progress for many other species. Once genome sequences are known, genome-wide scans can be used to create lists of genetic differences between the extinct species and their closest living relatives (see [10], for example), which then become the initial targets for genome editing.

The successes of the Church lab and other groups demonstrate that genome editing using CRISPR/cas9 is feasible and efficient across a wide range of taxa [11]. The number of edits that would be required to turn, for example, an Asian elephant genome into a mammoth genome is not small; it is estimated that there are around 1.5 million nucleotide-level differences between these two species [10]. However, the number of edits can be minimized by replacing large pieces of the genome in a single edit or by focusing on changing only those genes that are phenotypically relevant. As links between genotype and phenotype remain largely unknown, in particular for non-model organisms, the capacity to engineer every change is likely to exist before we understand the function of every gene.

## Next steps

What happens after an extinct genome is resurrected is less clear. For mammoths, Asian elephants may be a suitable maternal host, but cloning by nuclear transfer has not yet been achieved for elephants [12]. For other species, cloning is less likely to be successful. If the closest living species is evolutionarily distant or considerably different in size from the candidate species for de-extinction, incompatibilities between the developing embryo and the surrogate mother may mean that alternative technologies, for example artificial wombs (ectogenesis), will need to be developed. Some species, including birds, cannot be cloned by nuclear transfer [13] and other methods, such as germ-line engineering, will have to be used for these species. After birth, these organisms will be reared in captive environments, which will require knowledge of each species’ welfare needs. Captive breeding may also have lasting consequences for behavior and physiology, which may affect the organism’s survival after release into the wild. As genome-engineering technologies advance to the stage where the first phase of de-extinction — birth — is feasible, the second stage — release into the wild — will be enabled by ongoing work in conservation biology that aims to minimize the potentially negative consequences of captive breeding.

Organisms are, of course, more than just the sum of the nucleotides that make up their genome sequences. Embryos that are derived from engineered cells will be exposed to the developmental environment of a different species. Newborns will be raised in social groups that are necessarily different from those of their own species. They will be introduced to different habitats, will consume different diets, and will establish different microbiomes. All of these factors will influence phenotype, and these effects are likely to vary among species and environments. In summary, genome editing may someday create an organism whose genome sequence very closely matches that of an extinct species, but the organism that develops from those edited cells will not be the same as the organism that went extinct.

## A new tool for biodiversity conservation

While extinction is forever, there is little doubt that genome engineering can and will be used to resurrect extinct traits. While this aspect of de-extinction is not as headline-grabbing as the idea of resurrected mammoths or massive flocks of passenger pigeons, it is potentially the most important. Human population growth and increasing per capita consumption are the main drivers of extinctions in the present day [14]. Climate change, much of it driven by anthropogenic factors, is reshaping the distribution of habitats too quickly to allow species to adapt to the changes. As populations decline, species are increasingly threatened by secondary drivers of extinction, including disease and inbreeding. Genome engineering enables the reintroduction of lost genetic diversity, or the introduction of traits that evolved in related species, into species that are struggling to survive. Thanks to advances in genome sequencing and assembly, the growing databases of population genomic data from non-model organisms, and the application of genome engineering tools to link these non-model genotypes to phenotypes [15], genome engineering could prove to be an important new tool for conserving biodiversity that is not yet extinct.

## Abbreviations

CRISPR: clustered regularly interspaced short palindromic repeats

## References

[1] Yirka B. Researchers take another step in bringing back a wooly mammoth. http://phys.org/news/2015-03-wooly-mammoth.html.

[2] The great passenger pigeon comeback. http://longnow.org/revive/projects/passenger-pigeon/.

[3] Faris S. Breeding ancient cattle back from extinction. http://content.time.com/time/health/article/0,8599,1961918,00.html.

[4] Powell W (2014). The American chestnut’s genetic rebirth. Sci Am.

[5] Shapiro B (2015). How to clone a mammoth: the science of de-extinction.

[6] Wilmut I, Schnieke AE, McWhir J, Kind AJ, Campbell KH (1997). Viable offspring derived from fetal and adult mammalian cells. Nature.

[7] Beyhan Z, Iager AE, Cibelli JB (2007). Interspecies nuclear transfer: implications for embryonic stem cell biology. Cell Stem Cell.

[8] Folch J, Cocero MJ, Chesne P, Alabart JL, Dominguez V, Cognie Y (2009). First birth of an animal from an extinct subspecies (*Capra pyrenaica pyrenaica*) by cloning. Theriogenology.

[9] Shapiro B, Hofreiter M (2014). A paleogenomic perspective on evolution and gene function: new insights from ancient DNA. Science.

[10] Lynch VJ, Bedoya-Reina OC, Ratan A, Sulak M, Drautz-Moses DI, Perry GH (2015). Elephantid genomes reveal the molecular bases of woolly mammoth adaptations to the Arctic. Cell Rep.

[11] Chen L, Tang L, Xiang H, Jin L, Li Q, Dong Y (2014). Advances in genome editing technology and its promising application in evolutionary and ecological studies. Gigascience.

[12] Loi P, Saragusty J, Ptak G, Holt WV, Brown JL, Comizzoli P (2014). Cloning the mammoth: a complicated task or just a dream. Reproductive sciences in animal conservation: progress and prospects (Advances in Experimental Medicine and Biology).

[13] Yan L, Gao JS, Rui L, Lu YN, Wan ZY, Yu XX (2014). A review of strategies for producing chimeric birds. Avian Biol Res.

[14] Pimm SL, Jenkins CN, Abell R, Brooks TM, Gittleman JL, Joppa LN (2014). The biodiversity of species and their rates of extinction, distribution, and protection. Science.

[15] Bono JM, Olesnicky EC, Matzkin LM (2015). Connecting genotypes, phenotypes and fitness: harnessing the power of CRISPR/Cas9 genome editing. Mol Ecol.

